# Melatonin and endothelial cell-loaded alginate-fibrin hydrogel promoted angiogenesis in rat cryopreserved/thawed ovaries transplanted to the heterotopic sites

**DOI:** 10.1186/s13036-023-00343-x

**Published:** 2023-03-28

**Authors:** Melika Izadpanah, Azizeh Rahmani Del Bakhshayesh, Zahra Bahroudi, Abbas Majdi Seghinsara, Rahim Beheshti, Mahdi Mahdipour, Mahsa Rezaii Zarnaghi, Parisa Hassanpour, Narges Mardi, Reza Rahbarghazi, Ali Abedelahi

**Affiliations:** 1 Department of Anatomical Sciences, Faculty of Medicine, Tabriz University of Medical Sciences, Tabriz, 5166714766 Iran; 2 Department of Tissue Engineering, Faculty of Advanced Medical Sciences, Tabriz University of Medical Sciences, Tabriz, Iran; 3 Department of Anatomical Sciences, Faculty of Medicine, Tarbiat Modares University, Tehran, Iran; 4 Department of Veterinary, Shabestar Branch, Islamic Azad University, Shabestar, Iran; 5 Stem Cell Research Center, Tabriz University of Medical Sciences, Tabriz, Iran; 6 Department of Reproductive Biology, Faculty of Advanced Medical Sciences, Tabriz University of Medical Sciences, Tabriz, Iran; 7 Department of Clinical Biochemistry, Faculty of Medicine, Tabriz University of Medical Sciences, Tabriz, Iran; 8 Student Research Committee, Tabriz University of Medical Sciences, Tabriz, Iran; 9 Department of Applied Cell Sciences, Faculty of Advanced Medical Sciences, Tabriz University of Medical Sciences, Tabriz, Iran

**Keywords:** Ovarian tissue, Freezing, Cryopreserved/thawed ovarian transplant, Fibrin-alginate hydrogel, Angiogenesis

## Abstract

**Background:**

Ischemic niche can promote follicular atresia following the transplantation of cryopreserved/thawed ovaries to the heterotopic sites. Thus, the promotion of blood supply is an effective strategy to inhibit/reduce the ischemic damage to ovarian follicles. Here, the angiogenic potential of alginate (Alg) + fibrin (Fib) hydrogel enriched with melatonin (Mel) and CD144^+^ endothelial cells (ECs) was assessed on encapsulated cryopreserved/thawed ovaries following transplantation to heterotopic sites in rats.

**Methods:**

Alg + Fib hydrogel was fabricated by combining 2% (w/v) sodium Alg, 1% (w/v) Fib, and 5 IU thrombin at a ratio of 4: 2: 1, respectively. The mixture was solidified using 1% CaCl_2_. Using FTIR, SEM, swelling rate, and biodegradation assay, the physicochemical properties of Alg + Fib hydrogel were evaluated. The EC viability was examined using an MTT assay. Thirty-six adult female rats (aged between 6 and 8 weeks) with a normal estrus cycle were ovariectomized and enrolled in this study. Cryopreserved/thawed ovaries were encapsulated in Alg + Fib hydrogel containing 100 µM Mel + CD144^+^ ECs (2 × 10^4^ cells/ml) and transplanted into the subcutaneous region. Ovaries were removed after 14 days and the expression of Ang-1, and Ang-2 was monitored using real-time PCR assay. The number of vWF^+^ and α-SMA^+^ vessels was assessed using IHC staining. Using Masson’s trichrome staining, fibrotic changes were evaluated.

**Results:**

FTIR data indicated successful interaction of Alg with Fib in the presence of ionic cross-linker (1% CaCl_2_). Data confirmed higher biodegradation and swelling rates in Alg + Fib hydrogel compared to the Alg group (p < 0.05). Increased viability was achieved in encapsulated CD144^+^ ECs compared to the control group (p < 0.05). IF analysis showed the biodistribution of Dil^+^ ECs within hydrogel two weeks after transplantation. The ratio of Ang-2/Ang-1 was statistically up-regulated in the rats that received Alg + Fib + Mel hydrogel compared to the control-matched groups (p < 0.05). Based on the data, the addition of Mel and CD144^+^ ECs to Alg + Fib hydrogel reduced fibrotic changes. Along with these changes, the number of vWF^+^ and α-SMA^+^ vessels was increased in the presence of Mel and CD144^+^ ECs.

**Conclusions:**

Co-administration of Alg + Fib with Mel and CD144^+^ ECs induced angiogenesis toward encapsulated cryopreserved/thawed ovarian transplants, resulting in reduced fibrotic changes.

## Introduction

During the last few years, human societies have witnessed significant advances in cancer diagnosis and treatment. Despite these progresses, the global rate of cancer incidence is impressive with high-rate mortality [1]. It is suggested that therapeutic regimes can contribute to the reduction or removal of fertility in females due to prominent ovarian follicle atresia. Based on the histological examination, the density and size of follicles are diminished with the promotion of fibrotic changes and suppression of angiogenesis potential. These conditions may increase the possibility of premature ovarian failure (POF) in cancer patients [2, 3]. Regarding the fact that the maintenance of life quality is important in females following chemotherapy regimens, thus strategies should be directed toward the application of modalities with the potential to preserve and/or restore ovarian tissue function [4]. Approaches such as embryo freezing, oocyte freezing either in an immature or mature state, and whole ovarian tissue freezing have been used in females suffering from cancers. Notably, the superiority of each method is closely associated with the type of anaplastic changes, therapeutic regime, age of cancer patient, and individual’s health status [2]. In this regard, whole ovarian tissue cryopreservation/thawing and re-implantation have been applied because numerous primordial follicle germ cells are maintained intact during the procedure. This approach can be performed at different stages of the reproductive cycle even in children without the necessity to postpone the therapeutic program [5].

Despite the efficiency of the cryopreservation technique, follicular competence is significantly diminished after orthotopic or heterotopic transplantation [6]. The lack of appropriate blood supply and ischemic damage are the main issues in autograft transplantation, leading to poor follicular reserve, ranging from 60 to 95% [7–9]. Studies in rodents indicated the initiation of delayed angiogenesis into cryopreserved/thawed ovarian transplants at ectopic sites after 48 h in which mature and functional vascular systems are detectable after one-week post-transplantation [7–9]. To this end, the stimulation of angiogenesis and blood supplementation can be an effective strategy to reduce the detrimental effects of the cryopreservation/thawing procedure and ischemic changes after transplantation [6].

The promotion of *de novo* vessel formation into the supporting biomaterials and scaffolds is the main aim of tissue engineering approaches, resulting in the suitable integration of grafts with the local tissues [10]. To date, several biomaterials enriched with different growth factors, hormones, and antioxidant compounds have been used to accelerate the rate of vascularization and restore ovarian tissue function [8]. In recent decades, the application of several natural anionic polysaccharides such as alginate (Alg) has paved a way for the fabrication/synthesis of engineered tissue modules [11, 12]. Alg-based scaffolds can be solidified and shaped using different divalent cations to provide 3D encapsulated structures. Unfortunately, the lack of appropriate attachment motifs and prominent anionic charge limit the solo application of Alg in tissue engineering [13]. Therefore, Alg is commonly used in combination with other substrates to improve the physicochemical properties of final composites [13].

Fibrin (Fib), a plasma protein, can form a clot and polymeric network. Due to several motifs, Fib maintains the physical interaction of cells with the supporting extracellular matrix (ECM) and improves cell attachment within the engineered scaffolds, resulting in improved growth rate and suitable phenotype acquisition [14, 15]. It was suggested that Fib-based scaffolds exhibited appropriate biocompatibility, and biodegradability as Fib can be easily eliminated without residual toxicity. In Fib-based composites, degradation rate, porosity, and physicochemical properties depend on the concentration of fibrinogen and thrombin [14–16].

**Fig. 1:**
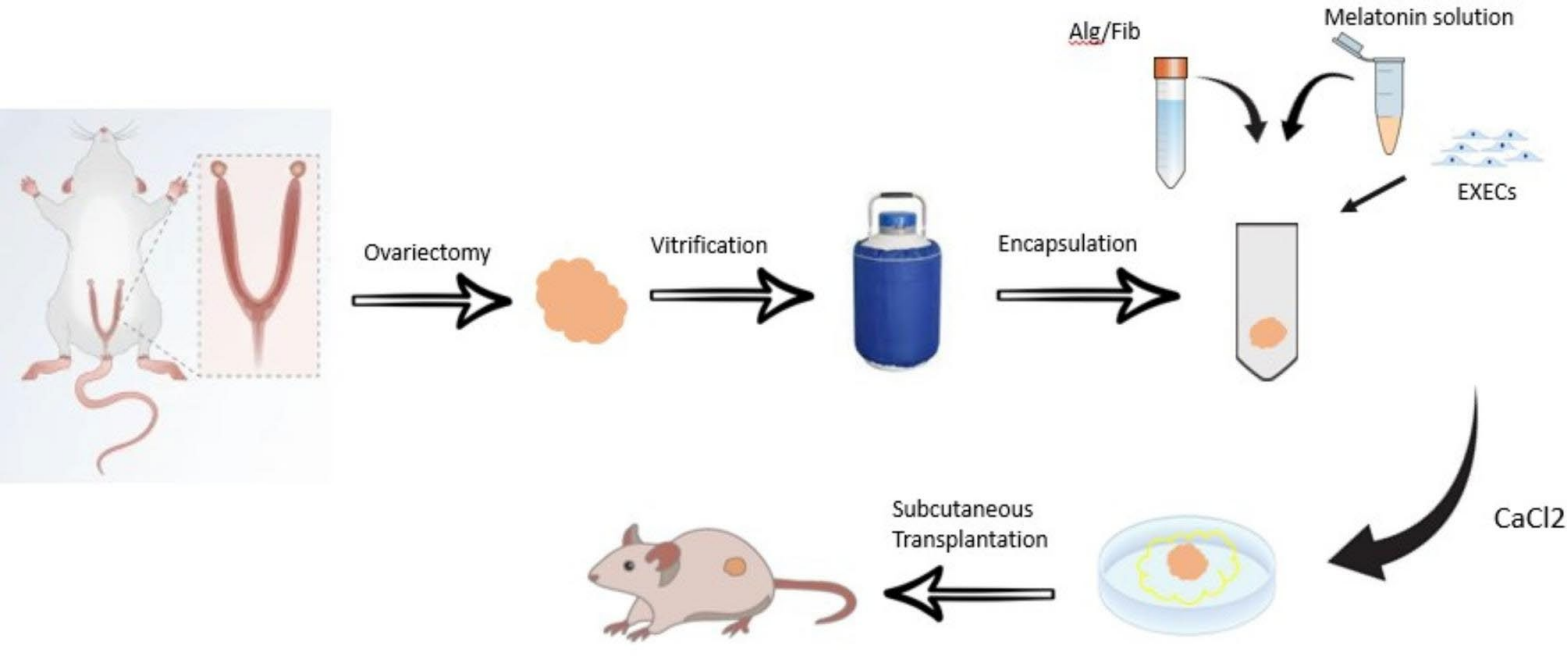
An illustration of the experimental procedure. Freshly isolated rat ovaries were sampled and cryopreserved using several solutions. Following embedding inside the Alg + Fib hydrogel with ECs, Mel, or their combination, ovaries were transplanted into the subcutaneous sites. Fourteen days post-transplantation, the angiogenesis status was monitored using proteomic and genomic analyses

In this study, the angiogenic potential of alginate (Alg) + fibrin (Fib) hydrogel enriched with melatonin (Mel) and CD144^+^ endothelial cells (ECs) was assessed on encapsulated cryopreserved/thawed ovaries following transplantation to heterotopic sites in rats (Fig. 1). It is proposed that the addition of Mel and CD144^+^ ECs can reduce toxicity and accelerate the formation of nascent blood vessels toward ovarian transplants.

## Methods

### Animals and ovarian tissue preparation

All steps of experimental protocols were confirmed by the local ethics committee of Tabriz University of Medical Sciences (IR.TBZMED.REC.1400.140) and rats were treated under the previously published guidelines (NIH, 1986). In the current study, 6-8-week-old female Wistar rats (weighing from 200 to 220 g) were housed at the animal house of Tabriz University of Medical Sciences under standard conditions (at 22 ± 2 °C, with a relative humidity of 50–60% and 12:12 light–dark cycle) with free access to water and chewing food. To obtain the ovarian tissues, rats were anesthetized using the combination of ketamine (80 mg/kg) and xylazine (10 mg/kg) via the i.p. route [17]. After that, the cutaneous tissue was disinfected and shaved at the ovarian region. Following the induction of a small lumbar incision, ovaries were sampled and subjected to subsequent experimental procedures. Here, rats were randomized into six different groups (each in 6) as follows; Control; rats transplanted with naked vitrified/thawed ovaries (FT); rats that received Alg-Fib hydrogel containing vitrified/thawed ovaries (FHT); rats transplanted with Alg-Fib hydrogel containing vitrified/thawed ovaries and Mel (FHT + Mel); rats transplanted with Alg-Fib hydrogel containing vitrified/thawed ovaries and ECs (FHT + ECs); and rats transplanted with Alg-Fib hydrogel containing vitrified/thawed ovaries, Mel plus ECs (FHT + Mel + ECs).

### Vitrification/thawing protocol

Samples were placed in different concentrations of equilibration and vitrification solutions for 5 min according to the following protocol [18]; **Solution I**: 5% ethylene glycol + 5% dimethyl sulfoxide + 20% fetal bovine serum (FBS) + 0.5 M sucrose dissolved in Dulbecco’s phosphate-buffered saline (DPBS); **Solution II**: 7.5% ethylene glycol + 7.5% dimethyl sulfoxide + 20% FBS + and 0.5 M sucrose dissolved in DPBS; **Solution III**: 10% ethylene glycol + 10% dimethyl sulfoxide + 20% FBS + 0.5 M sucrose dissolved in DPBS solution; **Solution IV**: 15% ethylene glycol + 15% dimethyl sulfoxide + 20% FBS + 0.5 M sucrose dissolved in DPBS solution. After the completion of these steps, ovaries were placed into cryotubes containing vitrification solution, exposed to nitrogen vapor (20 s), and stored at -196 °C for the next 15 min. Then, the samples were thawed at RT condition (for about 30 s) and kept inside the 37 °C water bath for 60 s. The protocol was continued with the ovarian tissue removal from cryotubes and suspending in descending concentrations of sucrose (1, 0.5, and 0.25 M dissolved in DPBS) for 5 min.

### Fabrication of alg + fib hydrogel

To this end, bovine plasma fibrinogen (Cat no: F8630; Sigma-Aldrich) and thrombin (Cat no: T7513 Sigma-Aldrich) dissolved separately in a normal saline solution containing 20 mM HEPES buffer and stored at -20˚C until use. To prepare sodium Alg, sodium Alg [MW: 70 kD; Kimica, Japan] was dissolved in calcium-free KRH buffer. The Alg + Fib hydrogel was prepared by combining 2% (w/v) sodium Alg, 1% (w/v) Fib, and 5 IU thrombin at a ratio of 4: 2: 1, respectively. The solution was mixed for 10 min to yield a homogenous solution followed by the addition of 1% CaCl_2_ (Cat no: 429,759; Sigma-Aldrich).

### Physicochemical properties

#### Degradability rate

The degradability of Alg + Fib hydrogel was calculated in phosphate-buffered saline (PBS) solution at 37 °C temperature after 1, 2, 3, and 4 days. At respective time points, hydrogels were sampled and dried. The mass loss % was calculated based on the following formula: Degradability rate (%) = (W_0 –_ W_t_)/ (W_0_) × 100. Where W_0_ stands for the hydrogel’s initial weight and W_t_ is the weight of hydrogels at respective time points.

#### Swelling capacity

The swelling capacity was monitored by placing hydrogel in PBS solution (pH = 7.4) at 37 °C after 1, 2, and 3 h. After completion of incubation times, samples were collected and excessive PBS was removed using filter papers. The swelling rates were calculated according to the following formula; Swelling capacity (%) = (W_2_-W_1_)/ (W_1_) × 100. W_1_ and W_2_ stand for initial hydrogel weight and weight of hydrogel at respective time points.

#### Scanning electron microscope (SEM) analysis

Hydrogel topography and surface morphology were visualized using SEM images. In this regard, hydrogels were frozen-dried and placed on an aluminum plate. After gold sputtering, hydrogels were analyzed using an SEM apparatus (Model: MIRA3 TESCAN; Czech Republic) at 15 kV.

### Enrichment of CD144^+^ ECs using MACS

To promote the angiogenesis capacity of Alg + Fib hydrogel, bone marrow CD144^+^ ECs were isolated from rat long bones using magnetic activated cell sorting (MACS). After the induction of euthanization using an overdose of ketamine and xylazine, femurs and tibias were exposed and placed in DPBS. Under sterile conditions, the muscles and adipose tissue remnants were excluded. The extremities were cut using sterile scissors and bone marrow content was aspirated into the 10 cm culture plates (SPL, South Korea) using syringes with 20 gauge needles. In the next step, mononuclear cells were purified by gradient density centrifugation using Ficoll-Hypaque solution (Cat no: F5415; Sigma-Aldrich). In brief, samples were diluted with DPBS at a ratio of 1: 1 and gently overlaid to the same volume of Ficoll-Hypaque solution. The samples were centrifuged at 300–400 g for 30 min and cells at the interphase were isolated and washed twice with DPBS. Freshly isolated cells were blocked with 1% bovine serum albumin (Cat no: A2153; Sigma-Aldrich) incubated with anti-CD144 microbeads (Order no: 130-123-932; Miltenyi Biotech; Germany) at 4 °C for 1 h. Using LS columns (Order no; 130-042-401; Miltenyi Biotech; Germany) and magnetic stands, CD144^+^ ECs were enriched and collected in separate tubes.

### MTT assay

To select the appropriate Mel concentration, we performed a conventional MTT assay on purified CD144^+^ ECs. To prepare Mel working solution, Mel (Cat no: M5250; Sigma-Aldrich) powder was dissolved in dimethyl sulfoxide solution. In the first step, 1 × 10^4^ CD144^+^ ECs/well of 96 well-plates were exposed to varied Mel concentrations (1, and 50 nm, 1 and 500 µM, and 1 mM) in 200 µl DMEM/HG culture medium supplemented with 1% FBS (Cat no: F2442; Sigma-Aldrich) and 1% Pen-Strep (Pen-Strep; Cat no: P4333; Sigma-Aldrich) and kept under standard conditions (at 5% CO_2_ and 37 °C). After 4 days, supernatants were discarded and 200 µl MTT solution (Dilution 3 mg/ml; Cat no: M5655) was overlaid to each well. After 3–4 h, the supernatants were discarded followed by the addition of dimethyl sulfoxide. The optical density was read at 570 nm and data were expressed as % of the control group. To see whether synthesized Alg + Fib hydrogel has no cytotoxic effects, the survival rate of CD144^+^ ECs was measured after 4 days. About 50 µl Alg + Fib hydrogel was poured into each well of 96-well plates and further gelled by the addition of 100 µl 1% CaCl_2_. A similar MTT protocol was used for the calculation of CD144^+^ EC viability as mentioned above. In this study, the final concentration of dimethyl sulfoxide was below 1%.

### CD144^+^ labeling

Using a CellTracker™ CM-DiI (Cat no: D3911; Invitrogen), CD144^+^ ECs were labeled before transplantation to the subcutaneous region. Cells were stained with 25 µM CM-DiI at 37 °C for 30 min. After three-time PBS washes, cells were used for in vivo studies. To monitor the distribution of cells within the hydrogel, 5 μm thick slides were prepared using cryosection after the completion of in vivo transplantation period. DAPI (1 µg/ml) was used to stain the nuclei. Slides were visualized using an Olympus microscope (Model: BX41; Japan).

### Autotransplantation of cryopreserved/thawed ovaries

The cryopreserved/thawed ovaries were encapsulated within 700 µl Alg + Fib hydrogel supplemented with 100 µM Mel and CD144^+^ ECs (2 × 10^4^ cells/ml of the hydrogel). Samples were subcutaneously transplanted into the lateral supra flanks. Fourteen days after transplantation, rats were euthanized and ovaries were subjected to several analyses.

### Real-time PCR analysis

The expression of angiopoietin-1 (Ang-1) and angiopoietin-2 (Ang-2) was measured using a real-time PCR technique (Table 1). The samples obtained from different groups were immediately frozen in liquid nitrogen and stored at -196˚C. RNA extraction was done by Trizol Reagent (Cat no: 302-001; Gene All) and reverse-transcribed to cDNA using an RNase-free DNase synthetase kit (Cat no: PP-410 S; Jena Bioscience). The PCR reaction was done using Corbett Life Science (Rotor-Gene 6000) System and Fast Start SYBR Green Master (Roche). Real-time PCR amplifications were performed using the following program: denaturation of cDNA (1 cycle at 94˚C for 10 min), amplification (40 cycles at 95˚C for 10 s, 60˚C for 35 s, and 72˚C for 20 s), and melting curve analysis (1 cycle at 60 to 95˚C with 1˚C/seconds). The 2^−ΔΔCT^ method was used to evaluate the quantity.

**Table 1:** Primers list

Gene	Forward	Reverse	Annealing (°C)
*ang-1*	5′-GCCACTTGAGAATTACATTGTGG-3′	5′-CGCGGATTTTATGCTCTAATCAACTG-3′	59
*ang-2*	5′-GTCTCCCAGCTGACCAGTGGG-3′	5′-TACCACTTGATACCGTTGAAC-3′	59
*gapdh*	5′-CTCTAAGGCTGTGGGCAAGGTCAT-3′	5′-GAGATCCACCACCCTGTTGCTGTA-3′	59

### Masson’s trichrome staining

The lack of angiogenesis can lead to progressive degeneration and fibrotic changes in ovarian transplants [19]. In this study, Masson’s trichrome staining was performed to monitor the deposition of type I collagen fiber within the parenchyma of cryopreserved/thawed ovaries after in vivo transplantation. On day 14, ovaries were fixed in 10% buffered formalin solution (Cat no: HT501128; Sigma-Aldrich,), and 5 μm thick sections were stained with Masson’s trichrome as previously described [19].

### Immunohistochemistry (IHC) staining

The angiogenesis potential of Mel- and CD144^+^ ECs-loaded Alg + Fib hydrogel was investigated using IHC staining. For this purpose, paraffin-embedded blocks were cut into 5-µm thick slides and rehydrated in ascending concentrations of alcohols. The endogenous peroxidase activity was inhibited using 3% (v/v) H_2_O_2_. The antigen retrieval process was performed by the incubation of slides in boiling sodium citrate buffer with a pH value of 6 for 20 min. The procedure was followed by overnight incubation samples with anti-α-SMA (dilution: 1:100; Dako, Denmark) and anti-vWF (dilution: 1:100; Dako, Denmark) antibodies. After three washes with PBS, an HRP-conjugated secondary antibody (EnVision + Dual Link System HRP kit; Dako) with DAB was used to visualize the immunoreactive foci. For semi-quantification analysis, 10 high-power fields were randomly examined in each slide and the mean vWF^+^ ECs and α-SMA^+^ vessels were compared to the control hydrogel-free frozen group (FT).

### Statistical analysis

Graph Pad Prism software (Ver. 8.4.3) was used to analyze all data (mean ± SD). Differences between the groups were evaluated using a one-way ANOVA with Tukey post hoc analysis. In this study, *p* < 0.05 was considered statistically significant.

## Results

### FTIR analysis

**Fig. 2:**
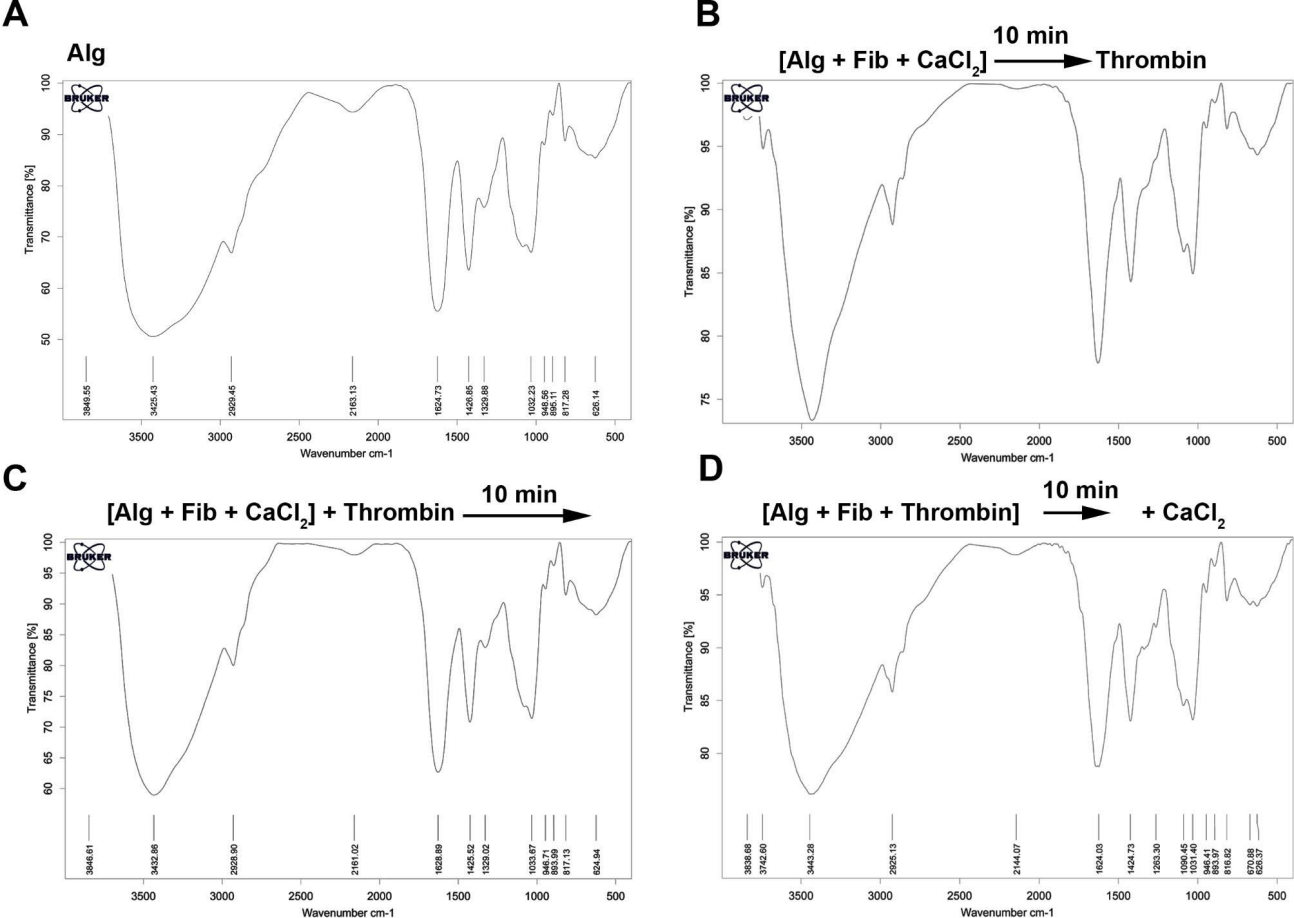
FTIR analysis of fabricated Alg + Fib hydrogels with different protocols was compared to the Alg alone group (**A-D**). In panel **B**, the mixture of Alg + Fib was mixed with CaCl_2_ for 10 min followed by the addition of thrombin. In another group (**C**), the mixture of Alg + Fib and CaCl_2_ was exposed to thrombin and allowed to gel for 10 min. In the last group (D), the mixture of Alg + Fib was clotted using thrombin and exposed to CaCl_2_ after 10 min. In all groups, 2% (w/v) sodium Alg, 1% Fib, and 5 IU thrombin were applied at a ratio of 4: 2: 1, respectively

FTIR analysis was performed to determine the interactions between the components within the polymeric network (Fig. 2A-D). Based on the data, the infrared spectra associated with Alg and Alg + Fib can be divided into two regions below 1700 cm^− 1^ and between 2500 cm^− 1^ and 4000 cm^− 1^. In the Alg spectra, the peaks at 1624 cm^− 1^ and 1032 cm^− 1^ are associated with the stretching vibration of carboxylate groups and the stretching vibrations of C-O bonds, respectively. The peak at 3425 cm^− 1^ corresponds to the O-H stretching vibration. Also, the peak at 2929 cm^− 1^ is related to the stretching vibration of C-H bonds (Fig. 2A-D). In the Alg + Fib group, peaks at 1624 cm^− 1^ (O = CNH), 1530 cm^− 1^ (N-H stretching vibrations), and 1263 cm^− 1^ (C-N stretching vibrations/N-H stretching vibrations) represent amide I, II, and III vibrations of fibrin, respectively. Also, peak 3443 cm^− 1^ indicates the interaction between the O-H and N–H groups (Fig. 2A-D). These peaks indicate the appropriate interaction of Fib with the Alg backbone in the final composite.

### Ultrastructural imaging

**Fig. 3:**
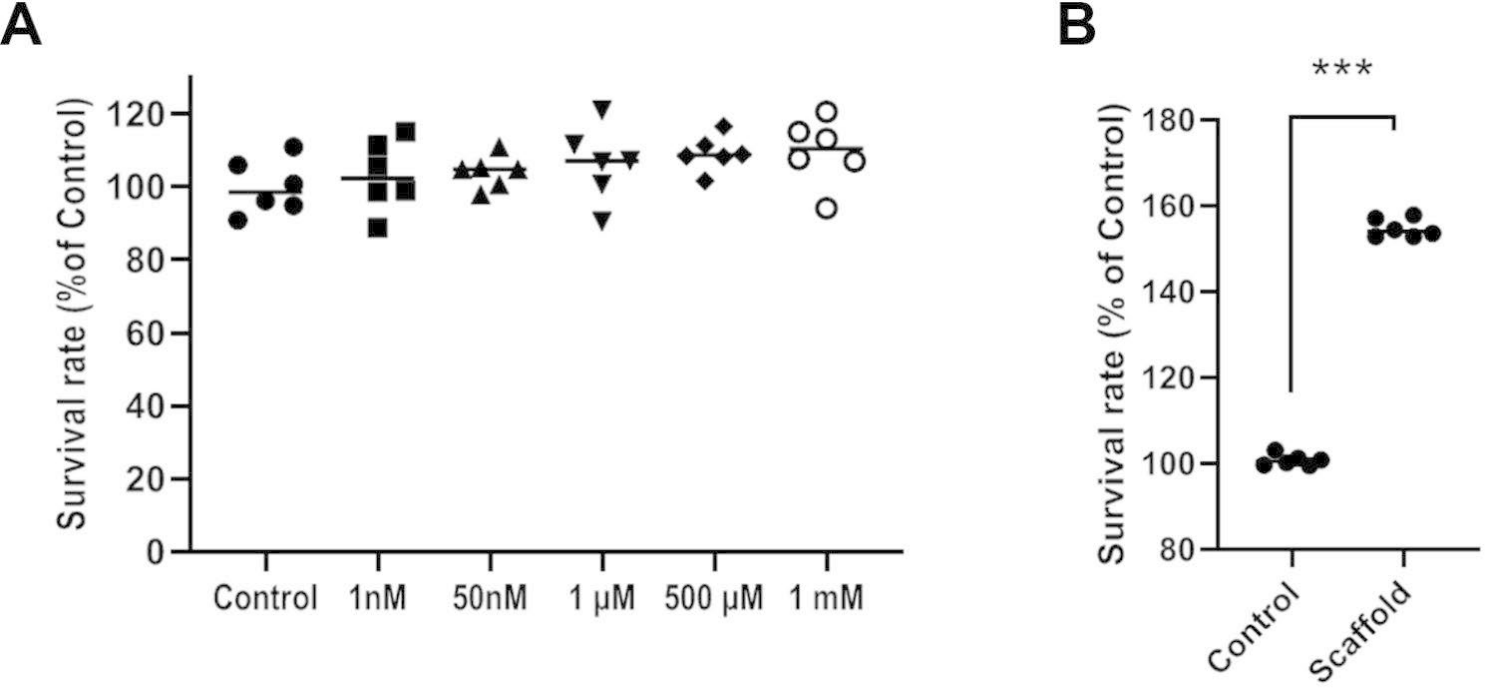
SEM images Alg + Fib (**A**). Ultrastructural imaging indicated the presence of cells within the cells (yellow arrows). Measuring the degradation rate over 4 days (**B**; *n* = 3). According to the data, the degradation rate was initiated 1 day after incubation in in vitro conditions without statistically significant changes. On days 2, 3, and 4, Alg + Fib at ratios of 1: 1, and 2: 1 exhibited a higher degradation rate compared to the Alg hydrogel alone. Swelling rate (**C**; n = 3). A higher swelling ratio was indicated in groups Alg + Fib at ratios of 1: 1, and 2: 1 compared to the Alg hydrogel alone. We found non-significant differences in terms of swelling rate between Alg + Fib (1: 1) and Alg + Fib (2: 1) at all-time points. One-Way ANOVA with Tukey post hoc. *p < 0.05; **p < 0.01; ***p < 0.001; ****p < 0.0001

Using SEM images, we monitored the structure of Alg + Fib hydrogel (Fig. 3A). Data indicated the presence of embedded ECs within the Alg + Fib hydrogel surface (yellow arrows).

### Degradation and swelling rates

It was noted that the degradation of all hydrogel types Alg, Alg + Fib (1: 1), and Alg + Fib (2: 1) was increased over time and reached maximum levels after 4 days (Fig. 3B). No statistical differences were obtained in terms of degradability during the first days. On day 2, Alg + Fib (1: 1), and Alg + Fib (2: 1) hydrogels were degraded fast compared to the Alg alone group (p < 0.05; Fig. 3B). According to the obtained data, the maximum mass loss percent is indicated in the Alg + Fib (1: 1) hydrogel compared to the other groups (p < 0.05; Fig. 3B). The Alg alone hydrogel exhibited less degradation rate in all time points compared to the Alg + Fib (1: 1), and Alg + Fib (2: 1) hydrogels. Compared to the Alg + Fib (2: 1) group, hydrogel composed of Alg + Fib (1: 1) had less degradation rate on day 2. However, these differences were not statistically significant on days 3 and 4. Similar to the degradation rate, we noted that all hydrogel swelled over time and maximum swelling rates were achieved after 3 h (Fig. 3C). Unlike the degradation rate, we found a non-significant difference in the swelling rate between the Alg + Fib (2: 1) and Alg + Fib (1: 1) hydrogels. Alg + Fib (2: 1) hydrogel exhibited a significant difference in swelling rate on day 4 as compared to Alg alone hydrogel while these values were significant in Alg + Fib (1: 1) group. According to these data, we selected Alg + Fib hydrogel at a ratio of 2: 1 for subsequent analyses.

### Survival rate

**Fig. 4:**
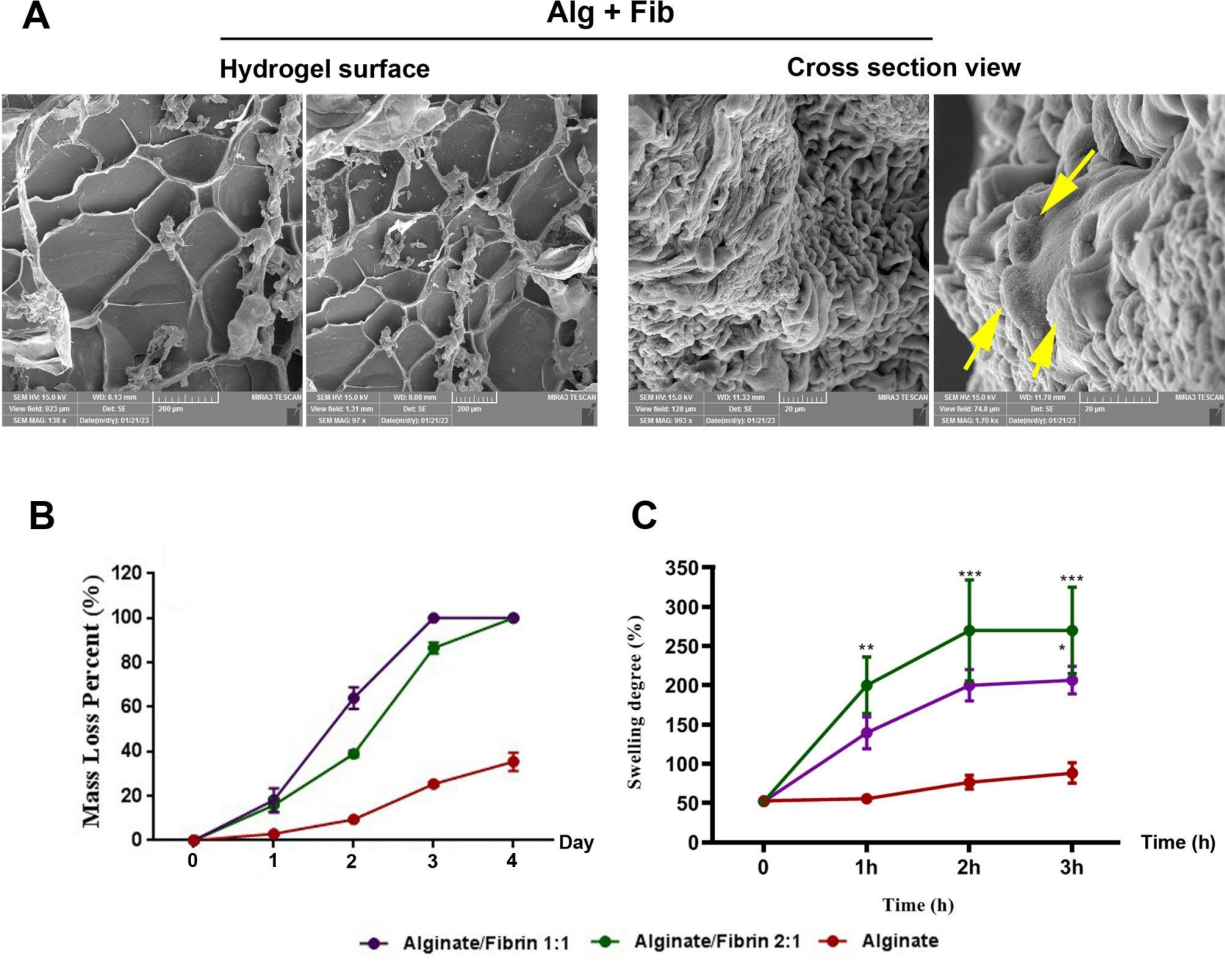
Measuring the survival rate of CD144^+^ ECs using MTT assay (**A-B**). Cells were exposed to different concentrations of Mel for 4 days (**A**; n = 6). Data revealed the lack of statistically significant differences in terms of cell viability compared to the non-treated control group (p > 0.05). The viability of CD144^+^ ECs was also calculated after being plated on Alg + Fib hydrogel after 4 days (**B**; n = 6). Data indicated an increase in cell viability compared to the cells plated on the plastic surface (p < 0.05). Student t-test. ***p < 0.001

MTT assay indicated that 4-day exposure of CD144^+^ ECs to ascending doses of Mel had no cytotoxic effects compared to the Mel-free control groups (p > 0.05; Fig. 4A). Despite a slight increase in the mean survival rate of groups treated with various Mel concentrations, these values did not reach statistically significant differences. In line with these data, we selected 100 µM Mel for subsequent *in vivo* analyses as previously described [20]. We also examined the cytoprotective properties of Alg + Fib hydrogel on CD144^+^ ECs after 4 days (Fig. 3). Data indicated an enhanced survival rate in CD144^+^ ECs plated on Alg + Fib surface compared to the CD144^+^ ECs cultured on the plastic surface (p < 0.05; Fig. 4B). These features demonstrate that Alg + Fib hydrogel provides a supportive niche for the induction of viability in rat CD144^+^ ECs.

### Cell tracking in vivo condition

**Fig. 5:**
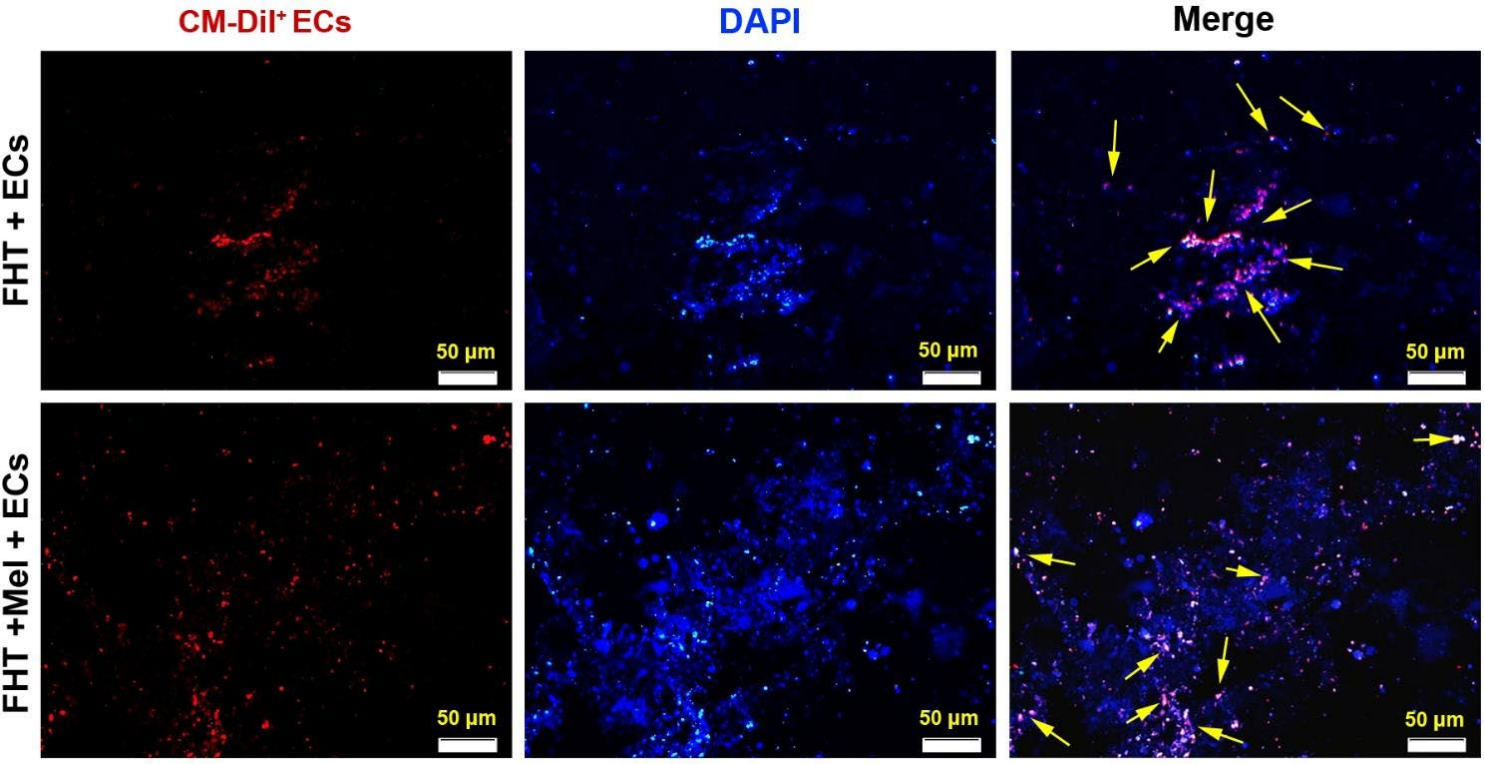
Tracking of CM-Dil labeled red CD144^+^ ECs 14 days after being transplanted into the subcutaneous site in a rat model. In FHT + ECs, and FHT + Mel + ECs groups, ECs are evident as red-colored cells distributed within the hydrogel (yellow arrows). Cell nuclei were stained with blue color DAPI.

Immunofluorescence images indicated numerous red-colored (CM Dil^+^ CD144 ECs) cells in FHT + ECs, and FHT + Mel + ECs groups after being transplanted into the subcutaneous region (yellow arrows; Fig. 5). Data confirmed that the DAPI^+^/CM Dil^+^ cells were evenly distributed within the gels although the presence of DAPI^+^ cells indicated the recruitment of rat host cells after 14 days (Fig. 5). Along with these data, the existence of DAPI^+^/CM Dil^+^ CD144 ECs within the transplanted Alg + Fib hydrogel indicated suitable cytocompatibility in in vivo conditions after 14 days.

### Real-time PCR analysis

**Fig. 6:**
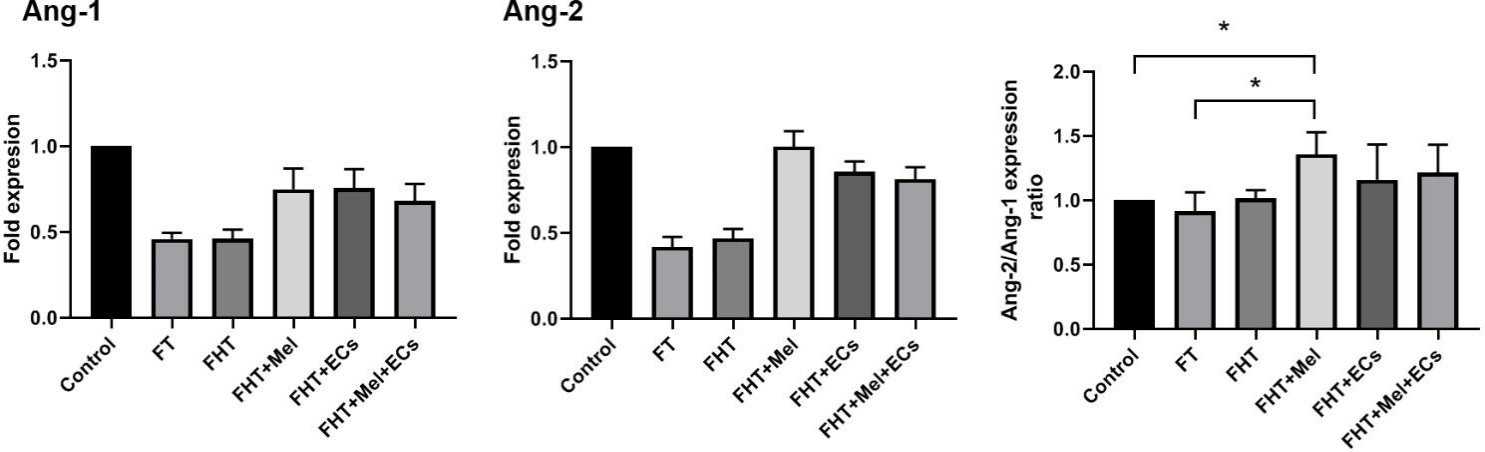
Monitoring the expression of angiogenesis-related genes Ang-1 and Ang-2 using real-time PCR after 14 days (n = 3). Data indicate increased, but non-significant, expression of Ang-1 and Ang-2 genes in FHT + Mel, FHT + ECs, and FHT + Mel + ECs groups compared to the FT and FHT groups. Data indicated an increased Ang-2/Ang-1 ratio in FHT + Mel group compared to FT and control rats. These levels did not reach significant levels in FHT + ECs, and FHT + Mel + ECs groups. One-Way ANOVA with Tukey post hoc. *p < 0.05

Data indicated that Ang-1 and Ang-2 were up-regulated in FT and FHT (Alg + Fib alone) rats compared to the non-transplanted control group (Fig. 6). We found that the transplantation of encapsulated cryopreserved/thawed ovaries with Mel, ECs, or their combination increased Ang-1 and Ang-2 expression compared to the FT and FHT groups. Despite the increase of Ang-1, changes did not yield statistically significant differences (Fig. 6). Similar to Ang-1, the maximum expression levels of Ang-2 were indicated in FHT + Mel compared to the control group. Despite marked increased expression, the levels of Ang-1 and Ang-2 were not statistically significant in FHT + Mel, FHT + ECs, and FHT + Mel + ECs groups. Of note, we found a significant increase in the Ang-2/Ang-1 ratio in the FHT + Mel group compared to the control (p < 0.05). Again, these ratios were not statistically significant in FHT + ECs, and FHT + Mel + ECs groups related to the control rats. These data indicated that transplantation of cryopreserved/thawed ovaries in Alg-Fib hydrogel with Mel, ECs, or their combination is an appropriate strategy to promote the expression of angiogenesis genes such as Ang-1 and Ang-2.

### Histological analysis of ovarian tissue fibrosis

**Fig. 7:**
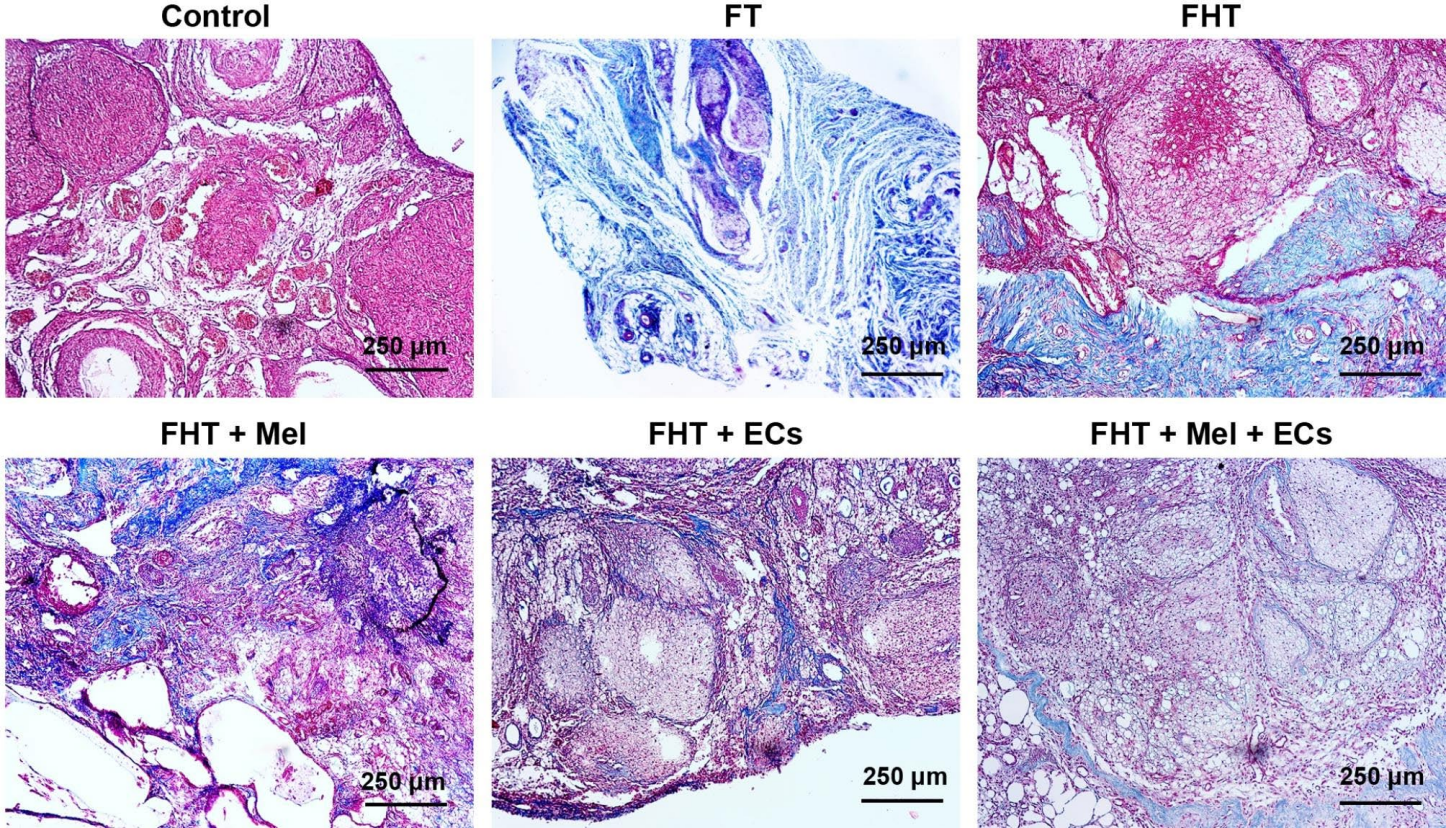
Masson’s trichrome staining of cryopreserved ovaries after 14 days of being transplanted into subcutaneous sites. In FT samples, bulk collagen fibers are present within the ovarian tissues and occupied the whole ovarian tissue section. In FHT, FHT + Mel, FHT + ECs, and FHT + Mel + ECs groups, the intensity of collagen fiber deposition was reduced. These changes were more evident in FHT + ECs and FHT + Mel + ECs groups. This assay was done in triplicate

Masson’s trichrome staining was performed to monitor fibrotic changes in cryopreserved/thawed ovaries after transplantation into the in vivo heterotopic site (Fig. 7). Bright-field imaging indicated the lack of detectable blue-colored collagen fibers within the parenchyma of freshly isolated ovaries. According to the data, transplantation of naked cryopreserved/thawed ovaries without supporting Alg + Fib led to the progressive fibrotic changes indicated by the deposition of collagen fibers (Fig. 7). Co-transplantation of cryopreserved/thawed ovaries with Alg + Fib hydrogel reduced slightly the intensity of fibrosis but still, the blue-colored collagen fibers can be detected in ovarian tissue parenchyma. Of note, the addition of Mel, ECs, or Mel + ECs reduced the intensity of collagen deposition. These features were more prominent in FHT + ECs, and FHT + Mel + ECs groups (Fig. 7).

### Co-transplantation of cryopreserved/thawed ovaries with Mel and ECs improved angiogenesis

**Fig. 8:**
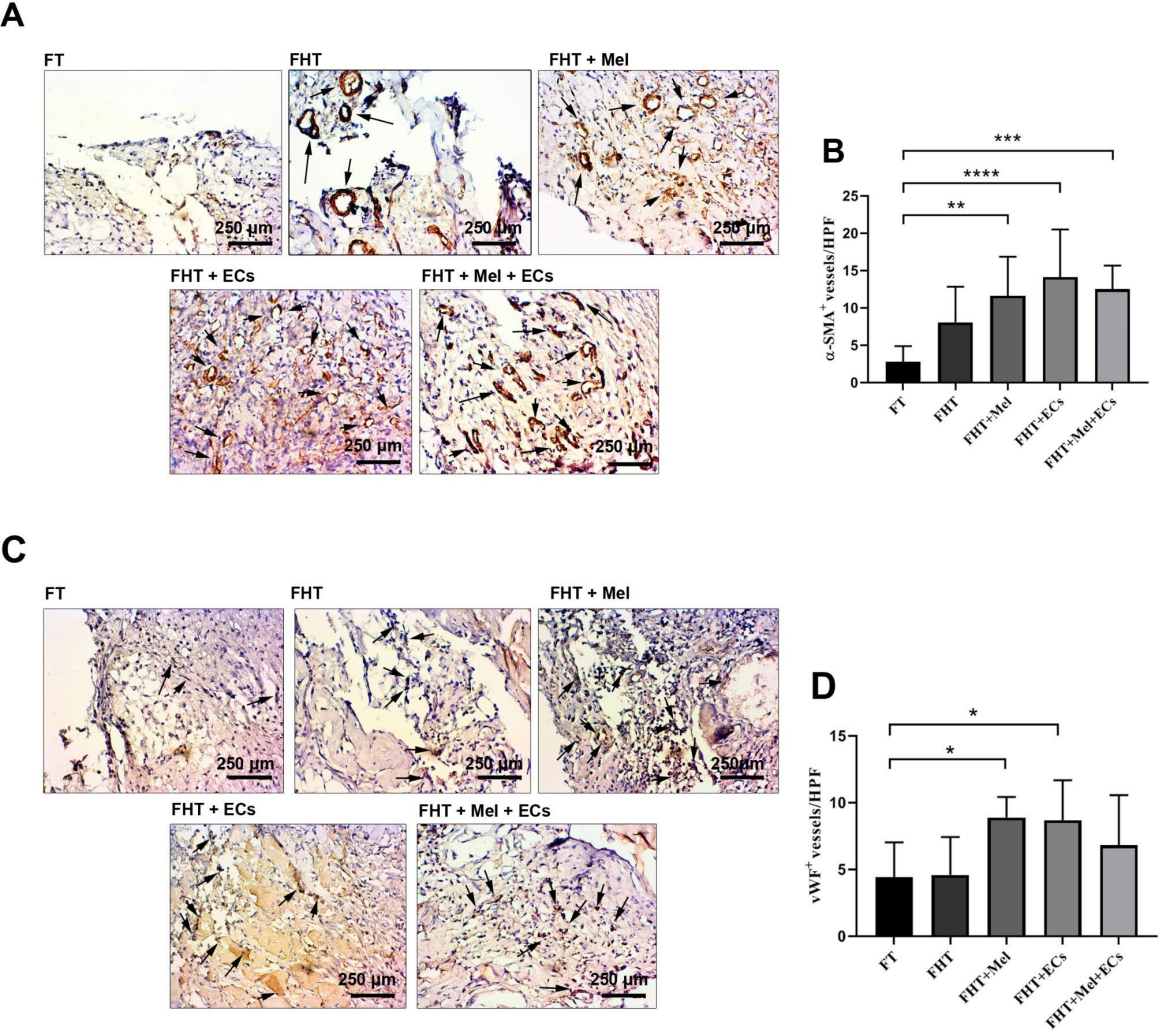
IHC staining for the assessment of vascular density (**A-D**; n = 10). The number of α-SMA^+^ vessels was increased in supporting hydrogels supplemented with Mel, ECs, and Mel + ECs compared to the FHT and FT groups (Black arrows; **A-B**). Along with these changes, the number of vWF^+^ vessels was also increased in FHT + Mel, and FHT ECs groups compared to the FT group (Black arrows; **C-D**). These changes did not reach statistically significant differences related to the FT and FHT groups. One-Way ANOVA with Tukey post hoc analysis. *p < 0.05; **p < 0.01; ***p < 0.001; ****p < 0.0001

Re-vascularization rate was assessed in different groups 14 days after transplantation of cryopreserved/thawed ovaries (Fig. 8A-D). To this end, protein levels of α-SMA (typical pericytes and smooth muscle cells marker) and vWF (EC marker) were monitored in prepared slides. Data indicated the non-significant increase of α-SMA in the FHT group compared to the FT group (p < 0.05; Fig. 8A-B). Co-transplantation of encapsulated cryopreserved/thawed ovaries with Mel, ECs, and Mel plus ECs led to an increase of α-SMA^+^ vessel density as compared to the FT group. According to our data, the maximum increase of α-SMA^+^ vessels occurred in FHT + ECs group. Similarly, we found statistically significant differences in FHT + Mel + ECs and FHT + Mel groups when compared to the FT group (p < 0.05; Fig. 8A-B). No statistically significant differences were found in terms of α-SMA^+^ vessels between the FHT + Mel, FHT + ECs, and FHT + Mel + ECs groups. Monitoring the density of vWF^+^ vessels indicated significant differences in FHT + Mel, and FHT + ECs, but not FHT + Mel + ECs groups compared to the FT groups. We also found non-significant changes in vWF^+^ vessel intensity between FT and FHT groups (p > 0.05; Fig. 8C-D). Similar to data from the α-SMA staining panel, the number of vWF^+^ vessels was not statistically changed between the FHT + Mel, FHT + ECs, and FHT + Mel + ECs groups. These data indicate that transplantation of cryopreserved/thawed ovaries with Alg + Fib hydrogel did not promote an efficient angiogenesis capacity in in vivo conditions while co-transplantation of Mel, ECs, and Mel + ECs can lead to higher vWF^+^ and α-SMA^+^ vessel intensities compared to the FT and FHT groups. Taken together, the angiogenesis capacity of Alg + Fib hydrogel is improved in the presence of Mel and ECs.

## Discussion

It is suggested that transplantation of cryopreserved/thawed ovaries to the heterotopic sites is an appropriate strategy to maintain fertility in individuals subjected to several insulting conditions [21]. It is thought that several issues such as the freezing method, ectopic transplantation site, and environmental factors can affect the functionality of cryopreserved/thawed ovarian cell events after transplantation [22]. To date, numerous attempts have been collected to improve the quality of ovarian transplants by the modulation of relevant factors. Based on the loss-of-function and gain-of-function experiments, angiogenesis is a fundamental procedure with the potential to reduce tissue hypoxic injuries, and fibrotic changes via the supplementation of oxygen and essential nutrients [6]. Emerging data point to the fact that scaffolds with distinct physiochemical properties are promising substrates in the maintenance of cryopreserved/thawed ovarian follicles with orthotopic and heterotopic re-transplantation [23]. Here, the angiogenic potential of Alg + Fib hydrogel enriched with Mel and CD144^+^ ECs was studied was assessed on encapsulated cryopreserved/thawed ovaries following transplantation to heterotopic sites in a rat model.

According to the FTIR analysis, Alg appropriately interacts with Fib in the presence of ionic cross-linker CaCl_2_. SEM images indicated porosity in Alg + Fib hydrogel. It is believed that these features can facilitate CD144^+^ EC homing and localization with appropriate morphological adaptation in in vitro and in vivo conditions [24]. Data indicated that Alg + Fib mixture exhibited higher degradation and swelling rates related to Alg alone hydrogel. Consistent with the present data, Deepthi and co-workers indicated that the interspersion of Alg nanobeads with Fib increased the swelling rate [25]. Since the high swelling rate allows the delivery of fluids and nutrients to the tissue and cells within the hydrogel structure, it can also increase the survival rate and blood vessel invagination. It was suggested that the formation of nascent blood vessels will be initiated days after the transplantation of cryopreserved/thawed ovaries in rodents [8]. Thus, it is vital to accelerate the process of angiogenesis and blood vessel formation using appropriate hydrogel and components to reduce the follicular atresia. It was noted that 4-day culture of CD144^+^ ECs on Alg + Fib hydrogel surface increased survival rate when compared to control cells plated on the plastic surface. Based on previous data, the mixture of Alg and Fib provides several adhesion motifs that promote attachment, and suitable morphology in mesenchymal stem cells and ECs, with enhanced survival and proliferation rates [26]. We also showed that encapsulated CD144^+^ ECs can distribute within the Alg + Fib hydrogel 14 days after being transplanted into the subcutaneous area.

It implies that ischemic injury in ovarian transplants can be reduced via the promotion of angiogenesis and blood supplementation [27, 28]. Angiopoietins like Ang-1 and − 2 participate in the latter steps of angiogenesis associated with vessel remodeling, and stabilization. In this regard, the secretion of Ang-1 via pericytes promotes vascular quiescence and their interaction with ECs leads to the maturation of newly generated vessels [29, 30]. In contrast, the elevation of Ang-2 with VEGF can loosen EC‒to‒EC connection and stimulate cell migration and angiogenesis properties [29]. Commensurate with these comments, the Ang-2/Ang-1 ratio is integral to EC activation and angiogenesis outcome [31]. Here, we found that the expression of Ang-1 and Ang-2 was increased non-significantly in encapsulated ovaries in the presence of Mel, CD144^+^ ECs, or Mel + ECs compared to the FT and FHT groups. According to our data, the Ang-2/Ang-1 ratio was statistically significant in FHT + Mel group compared to the FT and FHT groups. One reason for the lack of significant data in FHT + ECs and FHT + Mel + ECs groups would be that gene expression changes usually precede protein changes. That said, the angiogenesis procedure was possibly activated in these groups earlier in comparison with FHT + Mel group. IHC analysis revealed the promotion of angiogenesis and increase of vascular density, either vWF^+^ and α-SMA^+^ vessels, in encapsulated ovaries with Mel, ECs, and Mel + ECs. Along with our data, Limor and colleagues found that co-transplantation of Fib clot encapsulated ovaries with exogenous ECs improved organ function and competence in a mouse model [32]. It has been implicated that the recruitment of ECs to the periphery zone is a preliminary biological step that can accelerate the initiation of angiogenesis in grafts [32]. Whether and how exogenous and endogenous ECs participate in the promotion of angiogenesis needs further experiments. The addition of Mel was done with the respect to the fact that this hormone can blunt the detrimental effects of reactive free radicals in the early days after transplantation before the formation of *de novo* vessels [33, 34]. Besides, this hormone can increase the local content of pro-angiogenesis factors from ECs and immune cell origin [35]. According to our data, Mel- and ECs-free Alg + Fib hydrogels lack eligibility to induce angiogenesis as compared to the FT group. These features indicate that the addition of Mel and ECs is a strategy to promote angiogenesis deeper layer of graft hydrogel. In line with the promotion of angiogenesis, fibrotic changes were reduced in the presence of Mel, ECs and their combination. As the consequence, the current study illustrated that co-transplantation of Alg + Fib hydrogel enriched with ECs and Mel provides a significant benefit to ovarian tissue viability through resuming angiogenesis and diminishing fibrosis following the transplantation of cryopreserved/thawed ovaries in rats.

## Conclusions

In conclusion, we demonstrated that Alg + Fib hydrogel alone is not a suitable scaffold to promote angiogenesis and reduce ischemia-related degeneration after two weeks in in vivo conditions. Co-administration of Mel with suitable cell counterparts like endothelial lineages is an appropriate strategy to impede organ injury via the stimulation of vascularization.

## Data Availability

All relevant data will be freely available on a reasonable request.

## Abbreviations

Alg: Alginate
Ang-1, -2: Angiopoietin-1 and − 2
CaCl_2_: Calcium Chloride
DAB: 3,3’-diaminobenzidine
DAPI: 4′,6-diamidino-2-phenylindole
DPBS: Dulbecco’s phosphate-buffered saline
ECM: Extracellular matrix
ECs: Endothelial cells
FBS: Fetal bovine serum
Fib: Fibrin
HRP: Horseradish peroxidase
IHC: Immunohistochemistry
MACS: Magnetic activated cell sorting
Mel: Melatonin
OTT: Ovarian tissue transplantation
PBS: Phosphate-Buffered Saline
POF: Premature ovarian failure
SEM: Scanning Electron Microscope
vWF: von Willebrand factor
α-SMA: Alpha Smooth Muscle Actin
